# Liver tissue fragments obtained from males are the most promising source of human hepatocytes for cell-based therapies – Flow cytometric analysis of albumin expression

**DOI:** 10.1371/journal.pone.0182846

**Published:** 2017-08-09

**Authors:** Karolina Ewa Zakrzewska, Anna Samluk, Agnieszka Wencel, Krzysztof Dudek, Dorota Genowefa Pijanowska, Krzysztof Dariusz Pluta

**Affiliations:** 1 Department of Hybrid Microbiosystems Engineering, Nalecz Institute of Biocybernetics and Biomedical Engineering, Polish Academy of Sciences, Warsaw, Poland; 2 Chair and Department of General, Transplant and Liver Surgery, Medical University of Warsaw, Warsaw, Poland; Universita degli Studi Di Cagliari, ITALY

## Abstract

Cell-based therapies that could provide an alternative treatment for the end-stage liver disease require an adequate source of functional hepatocytes. There is little scientific evidence for the influence of patient’s age, sex, and chemotherapy on the cell isolation efficiency and metabolic activity of the harvested hepatocytes. The purpose of this study was to investigate whether hepatocytes derived from different sources display differential viability and biosynthetic capacity. Liver cells were isolated from 41 different human tissue specimens. Hepatocytes were labeled using specific antibodies and analyzed using flow cytometry. Multiparametric analysis of the acquired data revealed statistically significant differences between some studied groups of patients. Generally, populations of cells isolated from the male specimens had greater percentage of biosynthetically active hepatocytes than those from the female ones regardless of age and previous chemotherapy of the patient. Based on the albumin staining (and partially on the α-1-antitrypsin labeling) after donor liver exclusion (6 out of 41 samples), our results indicated that: 1. samples obtained from males gave a greater percentage of active hepatocytes than those from females (*p* = 0.034), and 2. specimens from the males after chemotherapy greater than those from the treated females (*p* = 0.032).

## Introduction

According to data available from the Centers for Disease Control and Prevention chronic liver disease and cirrhosis are the 12th leading causes of death in the United States (1999–2014) [1]. Liver transplantation is a viable treatment option for end-stage liver disease and acute liver failure. However, a shortage of donors reduces the advantages of this procedure. Every year, in the United States, of the 15 000 people waiting for a liver transplant, 10% die [2]. Procedures such as hepatocyte transplantation and use of bioartificial liver devices could represent feasible therapies bridging the time for whole organ transplantation or supporting the regeneration of the liver [3]. All these alternatives require an adequate source of functional hepatocytes.

Successful isolation of primary human hepatocytes remains a challenge and comparative studies need to be carried out in cells derived from different sources. The majority of published studies have focused on two aspects of the problem: cell yields and viability. Physiological activity or synthetic capacity of the isolated hepatocytes still pose a poorly recognized problem. It is commonly accepted that warm and cold ischemia time are one of the most important factors affecting hepatocytes isolation [4–7]. Type of the liver tissue (resected fragments, donor liver, diseased liver, cirrhotic liver, *etc*.) as well as preoperative blood tests and histopathology can also be a predictive factor for determining the likelihood of a successful isolation [6–11]. On the other hand, there is little scientific evidence for the influence of patient’s age, sex, and chemotherapy on the quality of the harvested hepatocytes. Moreover, published research results are conflicting [7,10–13].

In this paper, we present a comparative study on the impact of the age, sex, and chemotherapy on the yield and viability of the metabolically active hepatocytes. We used a method based on immunostaining of cells and flow cytometry. To our best knowledge, the method developed to estimate quality, in particular ability to produce albumin and α-1-antitrypsin, of the isolated hepatocytes by the flow cytometric analysis is published for the first time. Our results enabled us to rank sources of hepatocytes by the synthetic capacity of the isolated cells.

## Patients and methods

### Ethics statement

Liver tissue was obtained from surgical procedures carried out at the Department of General, Transplant and Liver Surgery, Medical University of Warsaw, Poland (October 2012 to March 2016). Ethical approval for the study was granted by the Local Research Ethics Committee (reference number KB/182/2008). Study was performed according to the principles of the 1975 Declaration of Helsinki. None of the transplant donors were from a vulnerable population and all donors or next of kin provided written informed consent that was freely given.

### Human liver cells isolation

Human liver cells were isolated from fragments of normal tissue obtained from hepatic resections carried out for metastatic disease (colon/ovaries/breasts), primary liver cancer or other reasons (usually benign tumors or another type of liver disease). Some of the isolations were performed using tissue fragments from livers not suitable for transplantation (D, donor liver).

Twenty six of 41 patients enrolled in the study had received perioperative chemotherapy (13 women and 13 men). Five different treatment regimens had been applied: FOLFOX4 (3 women and 3 men), FOLFOX6 (3 women and 2 men), FOLFIRI (2 women and 6 men), LF4 (3 women and 1 man), and XELODA (2 women and 1 man).

In 10 patients who had received chemotherapy (10/26) 6 of their livers showed signs of steatohepatitis, 2—signs of fibrosis, and 2—signs of steatosis. Additionally, two individuals (2/41) had diabetes. None of the patients had cirrhosis.

The type of liver tissue and the results of cells isolations are presented in Table 1.

**Table 1:** The characteristic of liver tissue used and the main results of liver cells isolations.

ID	Sex/Age	Liver type *	Chemotherapy	Viability [%]	Yield [10^6^/g]
H2-12	M/60	colon	Y	95.7	3.9
H3-12	M/72	colon	Y	91.8	12.0
H4-12	F/51	colon	Y	95.3	2.4
H5-12	M/72	other	Y	97.9	4.1
H6-12	M/73	colon	N	97.6	11.7
H7-12	F/39	other	N	97.5	7.8
H8-13	M/49	donor (D)	N	97.0	2.7
H9-13	M/62	colon	Y	97.2	10.6
H10-13	F/54	ovaries	Y	97.8	6.1
H11-13	F/54	breasts	Y	96.9	4.4
H12-13	F/61	colon	Y	97.6	3.1
H13-13	F/57	colon	Y	95.0	3.9
H14-13	M/40	colon	Y	99.1	1.3
H15-13	F/53	colon	Y	98.6	1.6
H16-13	F/67	other	N	99.5	3.7
H17-13	F/50	colon	Y	97.2	1.7
H18-13	M/59	colon	Y	94.3	4.7
H19-13	M/43	liver	N	98.7	3.4
H20-13	F/64	other	Y	97.6	1.6
H21-13	F/49	breasts	Y	97.0	3.2
H22-13	F/22	liver	N	99.3	2.3
H23-13	M/64	donor (D)	N	97.3	2.0
H24-13	M/21	liver	Y	99.2	4.8
H26-14	F/62	breasts	Y	99.9	6.3
H27-14	M/69	colon	N	99.2	4.6
H28-14	M/57	colon	Y	98.7	2.8
H29-14	M/60	colon	Y	99.0	1.4
H31-14	F/44	colon	Y	96.0	2.5
H32-14	M/42	colon	Y	97.7	2.8
H33-14	M/70	colon	Y	99.0	1.9
H34-14	M/75	liver	N	95.9	3.7
H35-14	M/65	donor (D)	N	96.4	2.7
H36-14	M/70	colon	Y	96.0	2.0
H37-15	F/34	other	Y	97.3	7.8
H38-15	M/61	donor (D)	N	92.0	1.3
H39-15	M/45	donor (D)	N	95.1	3.7
H40-15	F/62	colon	Y	97.2	1.1
H41-15	F/63	other	N	96.6	7.1
H42-15	M/61	colon	Y	97.2	1.3
H43-15	M/61	donor (D)	N	98.2	2.6
H44-16	M/24	other	N	95.4	1.4
n = 41	M = 24 F = 17	colon = 20 other than colon = 21	Y = 26 N = 15	**97.2** (91.8–99.9) **	**3.1** (1.1–12.0) **

* colon/ovaries/breasts—metastasis of colon/ovaries/breasts cancer to the liver; donor–liver not used for transplant; liver–primary liver cancer; other–cases which do not fit into mentioned above

** **median values** with the range (in parentheses)

The isolation of liver cells was carried out using a method based on enzymatic tissue disaggregation. Following resection, the fragments were immediately placed in a sterile sealed string bag containing ice-cold University of Wisconsin (UW–ViaSpan; DuPont Industrial Biosciences, Wilmington, DE, USA) solution and processed within 1 hour. In cases of donor liver fragments, the specimens have been kept in sterile UW solution on ice for several hours (less than 12 hours). The isolation started with the washing of liver fragments through the exposed vessels with ice-cold phosphate-buffered saline (PBS, without Ca^2+^ and Mg^2+^, pH 7.2; Lab Empire, Rzeszow, Poland) until all remaining blood was removed (flow rate 10 mL/min). Next, the tissue was minced with a scalpel in pre-warmed (37°C) 0.05% collagenase I (Life Technologies, Warsaw, Poland) solution in the William’s E Medium (Sigma-Aldrich, Poznan, Poland) and kept under constant agitation on a magnetic stirrer at 37°C for 1 hour. The obtained cell suspension was filtered through the cell strainer with 70 μm nylon mesh (Corning Inc., Riverfront Plaza, NY, USA). After centrifugation at 366xg (7 min, 4°C), the residual erythrocytes were destroyed by the incubation of the pellet in the red blood cell lysis buffer solution (155mM NH_4_Cl, 10mM NaHCO_3_, and 0.1mM EDTA; POCH, Gliwice, Poland) at 37°C for 5 min. Next, the cells suspension was centrifuged over a Percoll (GE Healthcare Bio-Sciences, Uppsala, Sweden) cushion (1.04 g/ml) at 366xg (20 min, 4°C) to remove cell debris. Finally, after a washing step, the cell pellet was resuspended in the Dulbecco’s Modified Eagle Medium (Sigma-Aldrich, Poznan, Poland) supplemented with 10% fetal bovine serum (FBS; Biological Industries, Kibbutz Beit-Haemek, Israel). Viable and dead cells were discriminated and counted using the 0.4% Trypan blue (Sigma-Aldrich, Poznan, Poland) staining with a hemacytometer.

### Flow cytometry

Populations of the cells isolated from liver tissue were analyzed using flow cytometry (FACS, fluorescence-activated cell sorting). The immunostaining of the cells was described elsewhere [14]. Briefly, cells were fixed with 4% formaldehyde and permeabilized using 0.1% Triton X-100 solution in PBS. Prepared cells (1 × 10^6^ per sample) were incubated with fluorophore-conjugated specific antibodies (Table 2) for 1 hour at room temperature. Samples were analyzed immediately after staining with FACSCanto II flow cytometer (Becton Dickinson, Warsaw, Poland). Single immunostainings were used for the assessment of hepatocytes’ biosynthetic capacity. Unstained specimens and cells incubated with appropriate isotype immunoglobulins (Table 2) were used as negative controls.

**Table 2:** Antibodies used in this study.

Type	Antibody	Dilution	Source
primary	goat α-human-albumin-FITC	1:150	Bethyl Laboratories, Inc., Montgomery, TX, USA (A80-129F)
	goat α-human-α-1-antitrypsin-FITC	1:750	Bethyl Laboratories, Inc., Montgomery, TX, USA (A80-122F)
	mouse α -human-CD45-PerCP	1:100	Becton Dickinson, Warsaw, Poland (345809)
	mouse α -human-CD14-APC	1:100	Becton Dickinson, Warsaw, Poland (345787)
isotype control	goat IgG-FITC	1:100	Abcam, Cambridge, UK (ab37374)
	mouse α -T-2 mycotoxin-APC-Cy7	1:100	Abcam, Cambridge, UK (ab46739)

Human hepatoma C3A (ATCC No. CRL-10741) and osteosarcoma HOS (ATCC No. CRL-1543) cell lines were used to verify the specificity of antibodies against hepatic markers. FACSDiva software (Becton Dickinson, Warsaw, Poland) was used for data acquiring and analysis.

### Patients and statistical analysis

Thirty five patients from whom the liver tissue samples were collected (Table 1; donor livers, n = 6, excluded from the statistical analysis) were divided into the following groups: males (**M**) of size of 18 patients, females (**F**) = 17, without chemotherapy (**Ch-**) = 9, with chemotherapy (**Ch+**) = 26, under 60 years old (**A60-**) = 18, 60 years old and older (**A60+**) = 17, and certain combinations of above-mentioned ones. The group of 4 patients was the smallest one used for comparison (**F Ch-**).

Data were analyzed using R version 3.2.2. R is a Free Software under the terms of the GNU General Public License [15]. The differences between two independent groups were analyzed using the Welch's two-sample t-test. For multiple comparisons the Benjamini & Hochberg correction procedure [16] was applied. In multivariate analysis the two-way Analysis of Variance (ANOVA) for linear model with interaction term was used. ANOVA results were subsequently confirmed using the Tukey HSD test [17]. The *p*-values lower than 0.05 were considered as statistically significant. The results are presented as the median values with the range for each variable.

## Results

### Populations of cells isolated from liver tissue

**Fig 1:**
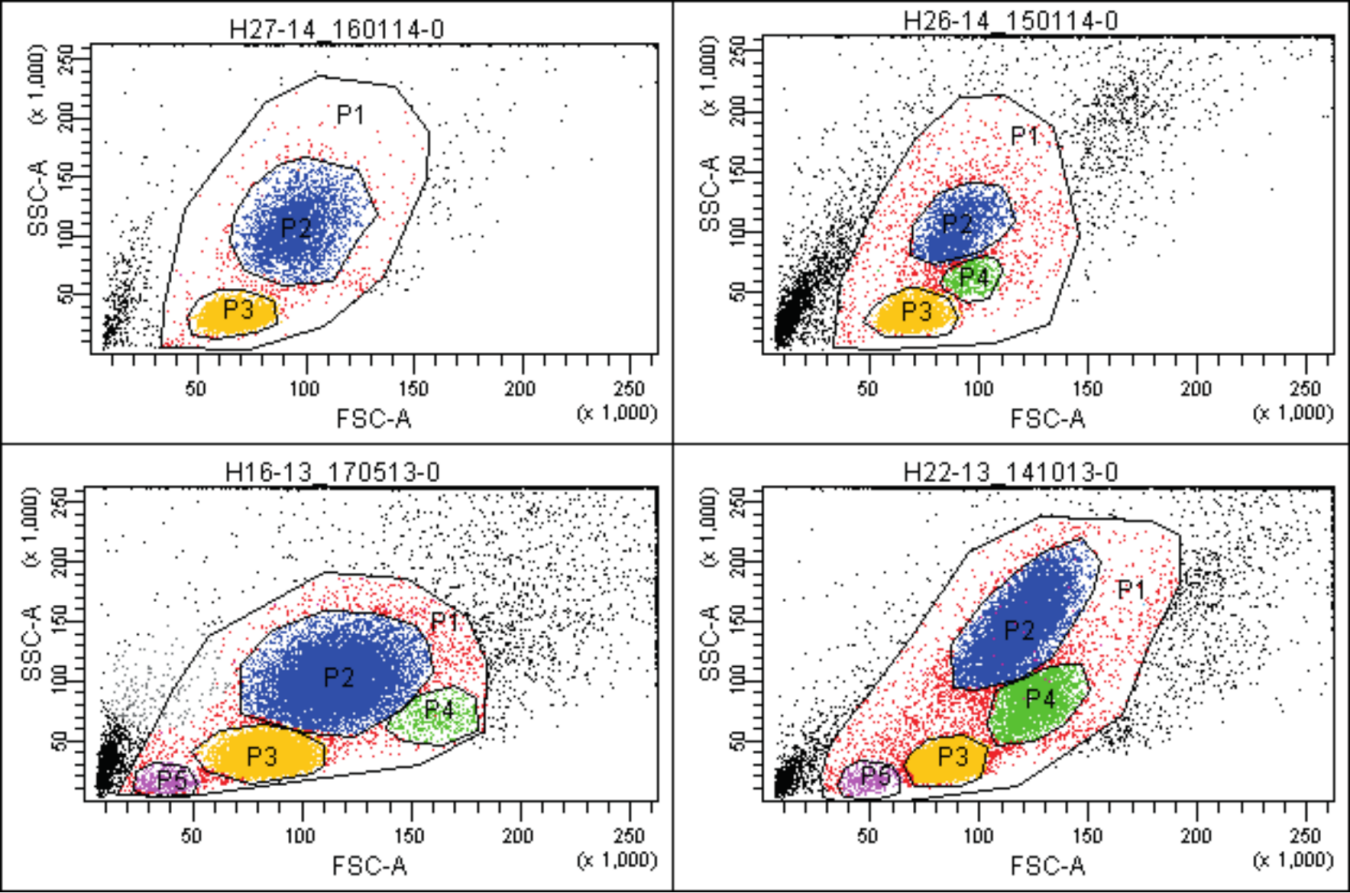
Populations of cells in liver isolates (light scatters). Exemplary flow cytometric dot plots showing populations of cells isolated from human liver tissue in light scatters: forward—FSC and side—SSC. Depending on specimen, there are minimum 2 and maximum 4 distinct cells populations (labeled P2 –P5) identified in the P1 gate (cellular debris and doublets excluded). Population P2 –blue dots; P3 –yellow dots; P4 –green dots; P5 –purple dots. The percentage of cells in the individual populations of the P1 gate—specimen H27-14: P2 = 35.9%, P3 = 62.8%; H26-14: P2 = 32.3%, P3 = 52.8%, P4 = 6.1%; H16-13: P2 = 47.6%, P3 = 46.4%, P4 = 1.7%, P5 = 1.3%; H22-13: P2 = 40.6%, P3 = 41.6%, P4 = 11%, P5 = 1.1%.

The rapid and low-stress method for the isolation of all cells from liver tissue gave high yields (**3.1** × 10^6^/g (1.1–12.0)) of viable cells (**97.2%** (91.8%–99.9%)) (Table 1). FACS analysis of the isolates using forward and side light scatter revealed the presence of minimum two and maximum four distinct cells populations: P2 –P5, depending on specimen (Fig 1).

**Fig 2:**
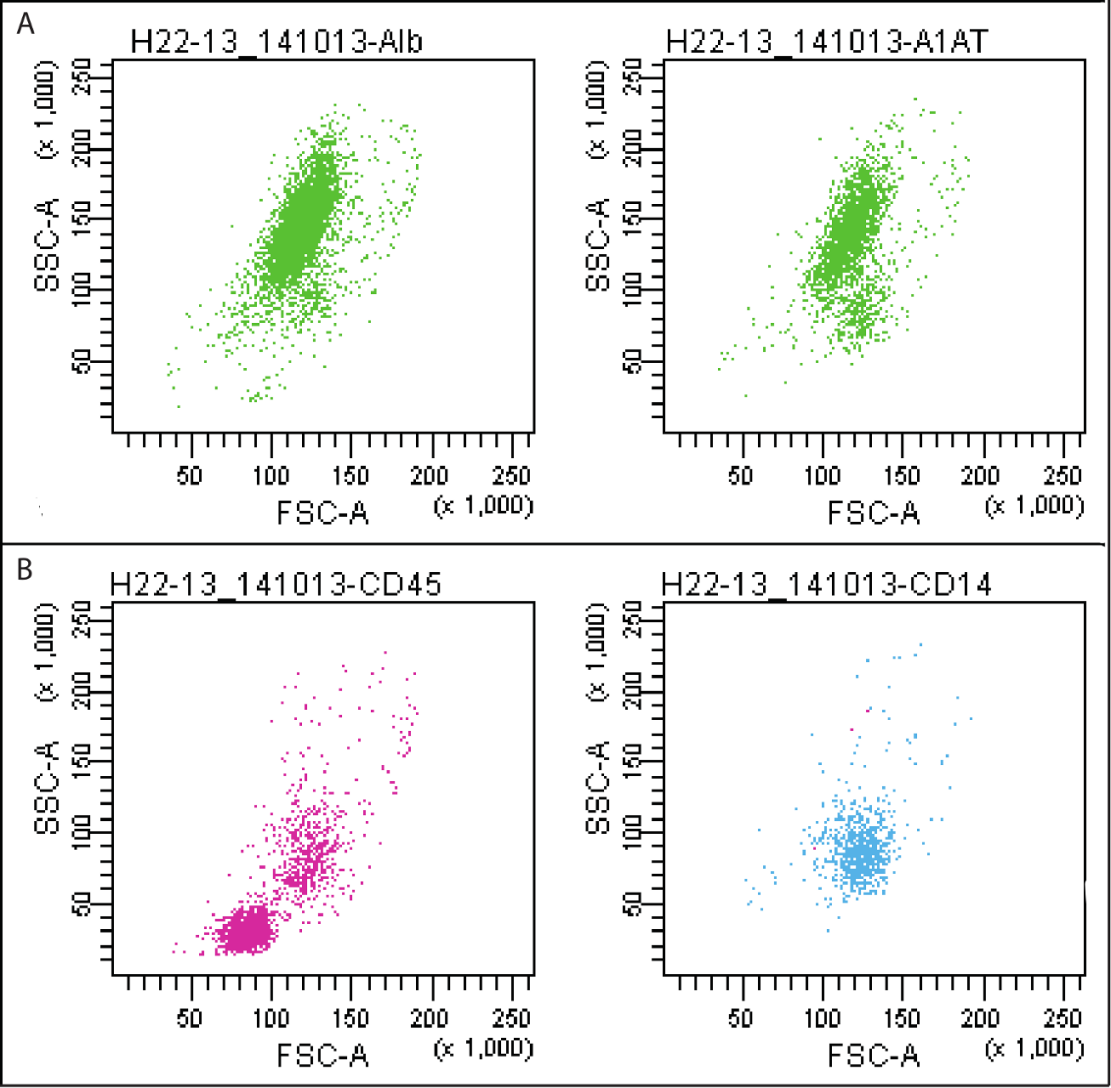
Staining of the isolated cells with fluorophore-conjugated specific antibodies. Exemplary immunostainings of the cells isolated from liver specimen (H22-13, see Table 1). (**A**) The cells labeled with anti-Alb (left panel) and anti-A1AT (right panel) antibodies were found predominantly in the P2 population (97.5% and 86.2% of the P2, respectively) and identified as hepatocytes. (**B**) Nonparenchymal liver cells were stained with anti-CD45 (populations P3 = 80.3% and P4 = 74.1%, left panel) and anti-CD14 antibodies (population P4 = 43.4%, right panel) (see Table 2 for the list of antibodies). Alb- and A1AT-positive cells–green dots; CD45-positive cells–pink dots; CD14-positive cells–blue dots. Abbr.: Alb, albumin; A1AT, α-1-antitrypsin; FSC, forward scatter; SSC, side scatter.

The hepatocytes are present in the P2. Nonparenchymal cells (liver sinusoidal endothelial cells, Kupffer cells, lymphocytes, biliary cells, stellate cells, various progenitors, etc.) belong to the other populations. The identity of the hepatocytes was confirmed by labeling with fluorophore-conjugated albumin (Alb)- and α-1-antitrypsin (A1AT)-specific antibodies (Fig 2A). The populations of cells other than P2 were labeled with anti-CD45 and anti-CD14 antibodies (Fig 2B).

**Fig 3:**
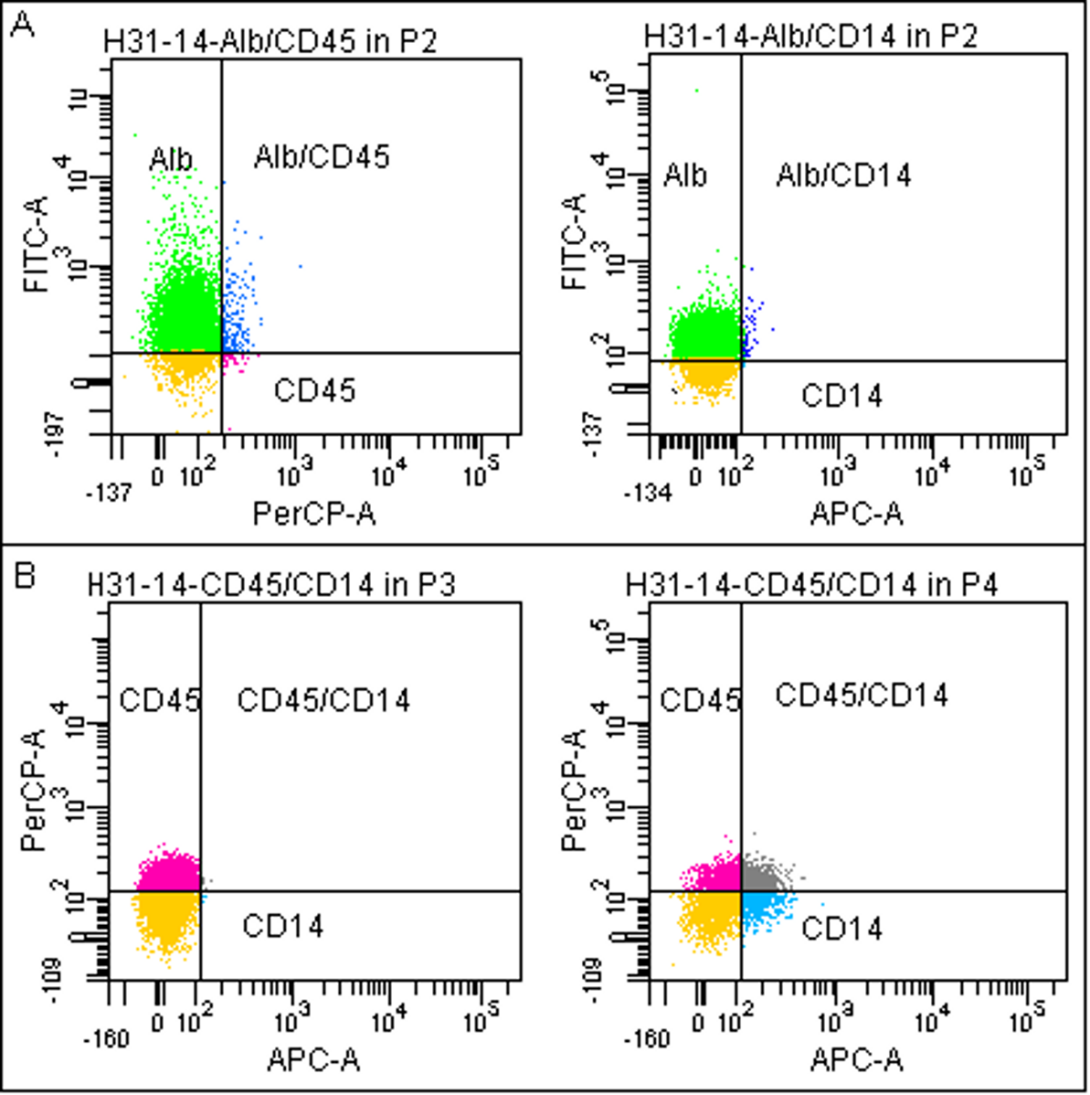
Double immunostaining of the isolated cells. Exemplary double immunostainings of the cells isolated from liver specimen (H31-14, see Table 1). (**A**) In the P2 population cells labeled with anti-Alb antibody expressed neither CD45 (83.5% vs.1.3%, left panel) nor CD14 (81.3% vs. 0.5%, right panel) markers. (**B**) Nonparenchymal cells in the P3 population (left panel) expressed CD45 marker but not CD14 (46.7% vs. 0.1%). Some of the cells from the P4 population (right panel) expressed both CD45 and CD14 markers (CD45 = 23.9%, CD14 = 16%, and CD45/CD14 = 15.1%) (see Table 2 for the list of antibodies). Alb-positive cells–green dots; CD45-positive cells–pink dots; CD14-positive cells–blue dots; CD54/CD14-positive cells–grey dots; unstained cells–yellow dots. Abbr.: Alb, albumin; FITC, PerCP, APC—fluorophores.

This staining is not sufficient to discriminate between different cell types populating liver but eventually allows for distinguishing parenchymal from nonparenchymal cells. As shown in Fig 3A, Alb-positive cells were labeled neither with anti-CD45 nor with anti-CD14 antibodies. On the other hand, some fractions of nonparenchymal cells exhibited immunoreactivity for both CD markers (Fig 3B). The CD45 and CD14 can be common markers for the Kupffer cells and monocytes/macrophages.

### Differential percentage of cells in P2 population

As expected, after the initial removal of most of the blood cells from the tissue fragment, hepatocytes represent a large fraction of the isolated cells. A median percentage of cells belonging to the P2 population in all samples (n = 41) was **47.6**. There were differences between groups observed in the entire patients’ population (see Patients and Methods section for description of cohorts), yet the statistically significant difference (*p* = 0.002) was observed only between donors (n = 6) and non-donors (n = 35) groups (**64.1%** (55.2%–86.2%) and **38.5%** (14.6%–81.2%), respectively). This above-average percentage of the P2 cells in the isolates from the donor livers is probably resulting from the different procedure applied for the pre-preparation of the whole donor organs. Nevertheless, the donor group was omitted from further statistical analysis, because it is not representative of the entire patients’ population (males only) and we found this tissue not suitable for hepatocytes’ isolation (this issue is discussed in the following section).

Generally, samples obtained from females (**F** = 17) gave greater percentage of the cells belonging to the P2 population then those from males (**M** = 18) (**47.6** (22.0–81.2) and **32.9** (14.6–75.3), respectively; *p* = 0.162), from people with no chemotherapy (**Ch-** = 9) greater than from treated (**Ch+** = 26) patients (**40.6** (18.8–75.3) and **35.1** (14.6–81.2), respectively; *p* = 0.823), and from younger people (**60-** = 18) greater than from older ones (**60+** = 17) (**48.6** (18.7–81.2) and **32.3** (14.6–75.2), respectively; *p* = 0.186). The two-way ANOVA model detected no interactions between above variables. The broad range of the percentage of cells found in the P2 population (14.6–81.2) is notable and suggests that these outcomes can be affected by both the quality of the tissue and by the effectiveness of blood removal (variable size of the P3–P5 populations). Thus, the meaning of these results is not clear and is not further discussed.

### Production of albumin and α-1-antitrypsin by the hepatocytes

Since the production of Alb and A1AT is a commonly accepted measure of metabolic capacity and viability of hepatocytes we have focused on these two markers. When all tissue samples were taken into account (n = 35), a median percentage of Alb-positive cells in the P2 population was **85.8** (56.5–98.7) and A1AT-possitive (data available for 29 samples: H9-13 – H44-16)– **88.9** (30.4–97.1).

#### Impact of sex and chemotherapy

**Fig 4:**
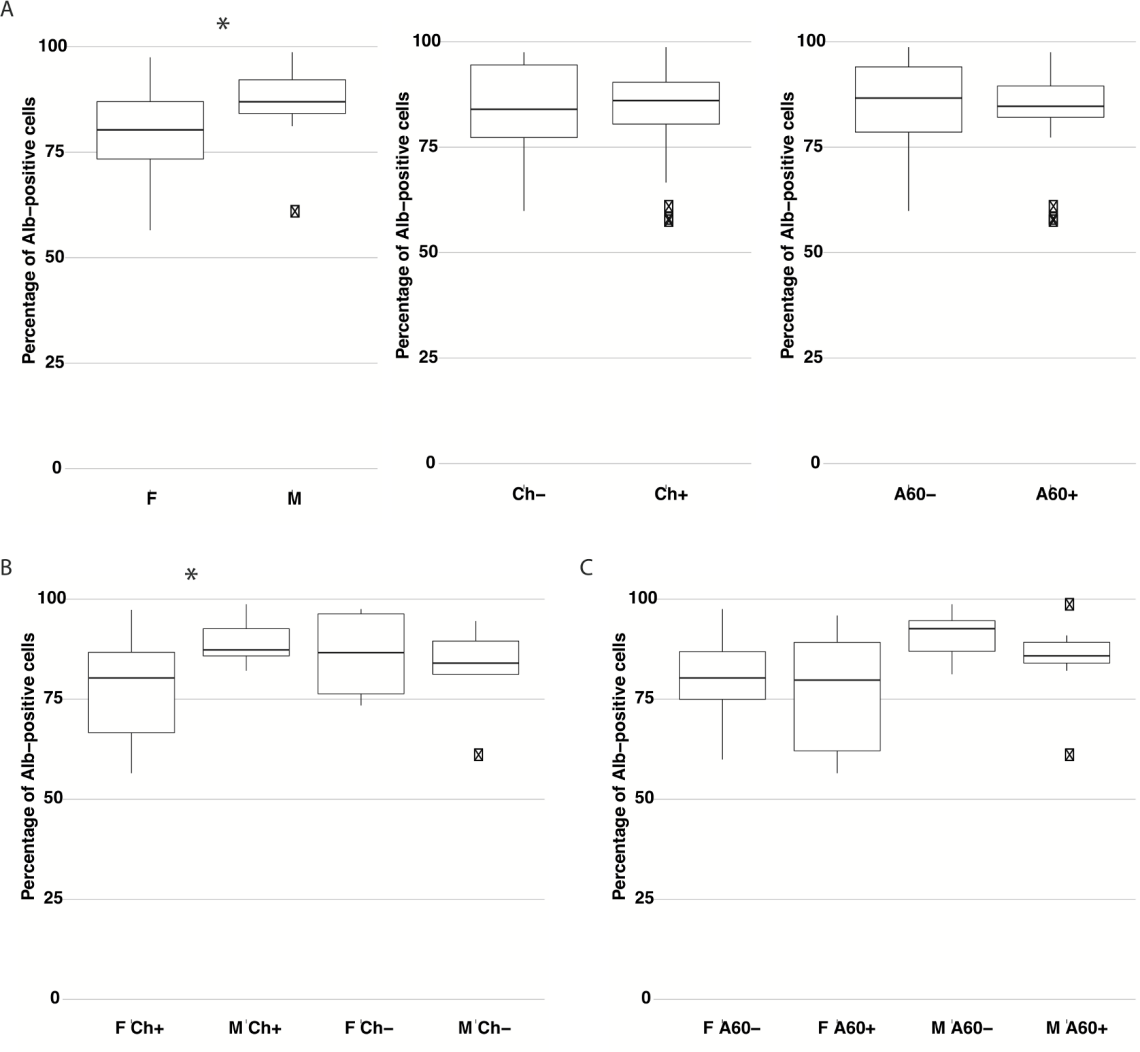
The impact of sex, chemotherapy, and age on the percentage of Alb-positive cells in P2 population. Comparison of the percentage of Alb-positive cells in the P2 population between groups of patients. (**A**) F (n = 17), M (n = 18); Ch- (n = 9), CH+ (n = 26); A60- (n = 18), A60+ (n = 17). (**B**) F Ch+ (n = 13), M Ch+ (n = 13), F Ch- (n = 4), M Ch- (n = 5). (**C**) F A60- (n = 11), F A60+ (n = 6), M A60- (n = 7), M A60+ (n = 11). The differences between two independent groups were analyzed using the Welch's two-sample t-test. For multiple comparisons the Benjamini & Hochberg correction procedure was applied. In multivariate analysis the two-way Analysis of Variance (ANOVA) for linear model with interaction term was used. ANOVA results were subsequently confirmed using the Tukey HSD test. The results are presented as the median values with the range for each variable. * *p* < 0.05 Abbr.: Alb, albumin; F, female; M, male; Ch-, non-treated patients; Ch+, patients after chemotherapy; A60-, patients under 60 years old; A60+, patients 60 years old and older.

In the single variable comparison, the difference in the percentage of Alb-positive hepatocytes in the P2 population between males (**M** = 18: **86.9** (59.9–98.7)) and females (**F** = 17: **80.3** (56.5–97.5)) was statistically significant (***p*** = 0.038) (Fig 4A, left panel). Interestingly, it seems that chemotherapy did not affect the percentage of Alb-positive hepatocytes when the entire studied population was analyzed (**Ch**- = 9: **84.0** (59.9–97.5) and **Ch+** = 26: **86.1** (56.5–98.7), ***p*** = 0.902) (Fig 4A, middle panel). However, when the groups of males and females with or without chemotherapy were compared, the impact of chemotherapy was apparent (***p*** = 0.04 after correction) between **M Ch+** = 13 (**87.3** (82.1–98.7)) and **F Ch+** = 13 (**80.3** (56.5–97.3)), but not significant (***p*** = 0.61 after correction) between non-treated patients: **M Ch-** = 5 (**84.0** (59.9–94.5)) and **F Ch-** = 4 (**86.6** (73.4–97.5)) (Fig 4B). Additionally, it appeared that chemotherapy had no effect in the patients within the same sex groups (**M Ch+**/**M Ch-**: ***p*** = 0.40 and **F Ch+**/**F Ch-**: ***p*** = 0.40).

In order to investigate the exact interactions between these two variables the two-way ANOVA test was performed. The *p*-value for the interaction term “M/F” was significant (0.034), whereas for term “Sex/Chemotherapy” it was not (0.058). However, it is close to the significance level and the interaction plot (S1 Fig) clearly reveals non-parallel lines, which indicates possible interaction between these variables. The ANOVA results were then confirmed using the Tukey HSD test. The *p*-value for the difference between males and females was 0.034 and for **M Ch+** and **F Ch+** groups– 0.032 (Table 3).

**Table 3:** The *p*-values for selected interactions from two-way Analysis of Variance (ANOVA) confirmed using Tukey HSD test.

Chemotherapy	
Ch-/Ch+	0.973
M/F	**0.034**
Sex/Chemotherapy	0.058
M Ch+/F Ch+	**0.032**
Age	
A60-/A60+	0.239
M/F	**0.039**
Sex/Age	0.772
M A60-/F A60-	0.234

Abbr.: F, female; M, male; Ch-, non-treated patients; Ch+, patients after chemotherapy; A60-, patients under 60 years old; A60+, patients 60 years old and older

#### Impact of age and sex

The age of the patients analyzed individually had no effect on the percentage of Alb-positive cells in the P2 population: **A60**- = 18 (**86.7** (59.9–98.7)) and A60+ = 17 (**84.7** (56.5–97.5)), ***p*** = 0.547 (Fig 4A, right panel). The difference was visible for **M A60-** = 7 (**92.6** (81.2–98.7)) and **M A60+** = 11 (**85.8** (59.9–97.5)), yet this difference was not statistically significant (***p*** = 0.42 after correction). Such a difference was not noticeable for females (**F A60-** = 11: **80.3** (59.9–97.5) and **F A60+** = 6: **79.8** (56.5–95.9), ***p*** = 0.52 after correction), but was considerable (although not statistically significant–***p*** = 0.19 after correction) between **M A60-** and **F A60-**. In the group of **A60+** patients (n = 17) the difference between males and females was smaller and not significant (***p*** = 0.32 after correction) (Fig 4C). In the multivariate analysis, the two-way ANOVA test again showed significant interaction for “M/F” (***p*** = 0.039 after Tukey HSD test) and no interaction between the sex and the age of the patients (***p*** = 0.772) (Table 3).

**Fig 5:**
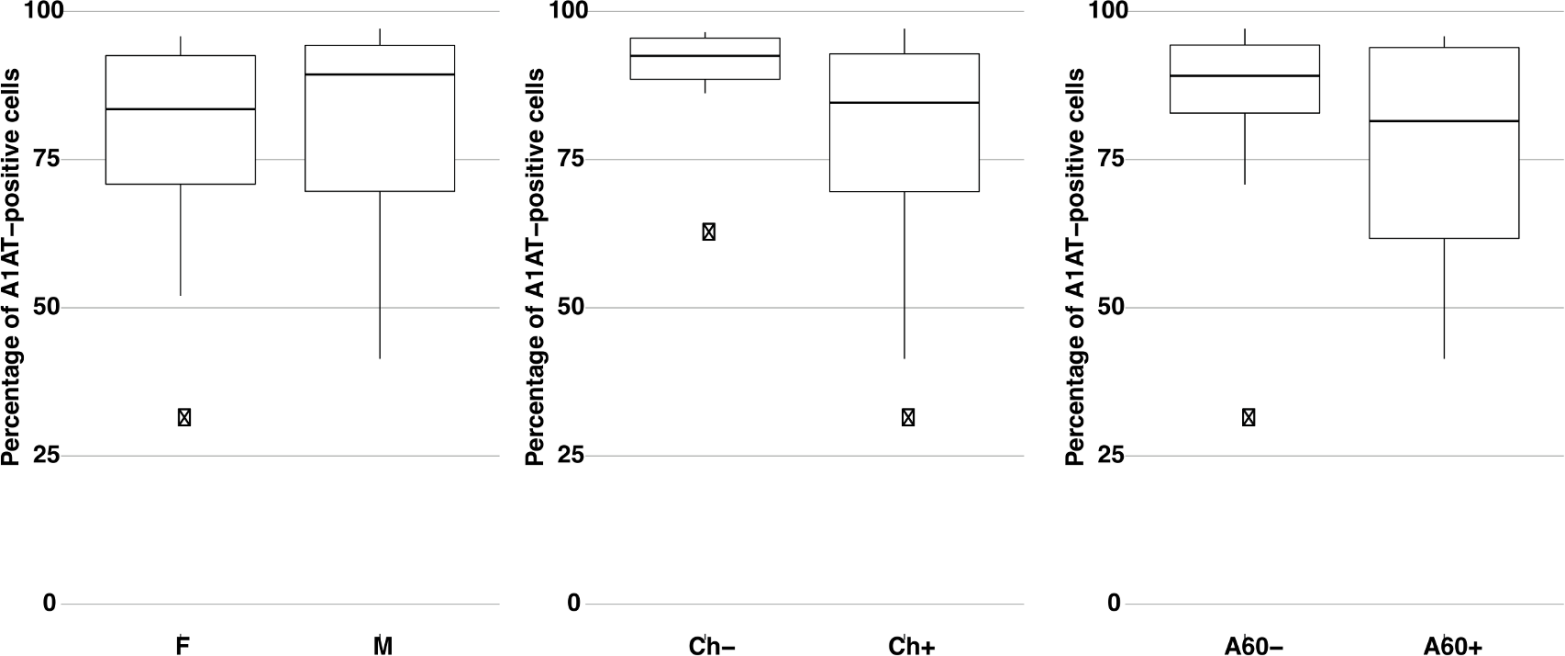
The impact of sex, chemotherapy, and age on the percentage of A1AT-positive cells in P2 population. Comparison of the percentage of A1AT-positive cells in the P2 population between groups of patients. F (n = 15), M (n = 14), Ch- (n = 7), Ch+ (n = 22), A60- (n = 16), A60+ (n = 13). The differences between two independent groups were analyzed using the Welch's two-sample t-test. The results are presented as the median values with the range for each variable. Abbr.: A1AT, α-1-antitrypsin; F, female; M, male; Ch-, non-treated patients; Ch+, patients after chemotherapy; A60-, patients under 60 years old; A60+, patients 60 years old and older.

Staining of the P2 cells with the A1AT antibody in part confirmed the results obtained for the Alb-positive cells. However, in the case of the percentage of the A1AT-positive cells, all three variables: sex, chemotherapy, and age analyzed individually seem to have no impact on the differences in studied groups. Some differences, even if visible, did not reach statistical significance: **M** = 14: **89.4** (41.4–97.1) and **F** = 15: **83.5** (30.4–95.8), *p* = 0.79; **Ch+** = 22: **84.6** (30.4–97.1) and **Ch-** = 7: **92.5** (61.7–96.5), *p* = 0.092; **A60-** = 16: **89.2** (30.4–97.1) and **A60+** = 13: **81.5** (41.4–95.8), *p* = 0.20 (Fig 5). Consequently, in multivariate analysis no statistically significant interactions were detected between these variables.

## Discussion

In order to simplify and hasten the procedure for liver cells isolation we have developed the method based on enzymatic tissue disaggregation. It provided high yields of cells, comparable to those presented in the available literature, with high viability [4–11,18,19]. However, this technique is rather appropriate for small tissue fragments (5–100 g) and does not include the step for hepatocytes enrichment. The classical and the most commonly used two-step collagenase perfusion, described for the first time by Seglen (1976) [20], is more suitable for the whole-organ or large-scale isolations. In spite of that, the presented method corresponds to the requirements of the flow cytometric analysis: fast and low-stress sample preparation. Specific isolation of the hepatocytes is not necessary since the desired cell population can be set apart using a cytometer.

We have observed only minor differences in both isolation efficiency and cells viability between analyzed groups. Neither age of the tissue donors, sex, and chemotherapy nor combinations of above-mentioned parameters gave *p*-values equal or lower than 0.05. In the study of criteria suitable for the identification of the most promising liver specimen Vondran *et al*.(2008) isolated human hepatocytes from tissue after partial hepatectomy (n = 50). As it was shown in our report, the patient’s sex and previous chemotherapy had no influence. Contrary to our results, donor age significantly affected the isolation outcome, but it was correlated with the overrepresentation of the primary and secondary tumors in the group of older patients. Thus, the quality of the individual specimen rather than the age was important and this parameter was not found suitable for predicting cell yields [11]. Also preoperative blood tests and histopathology (e.g. GGT levels, cholestasis, cirrhosis, and steatosis) should have a predictive potential, but in these fields results are conflicting [6–11]. Nevertheless, we have not analyzed the correlation between the indications for surgery and the outcome of isolation since there were no differences either in the cell yield (*p* = 0.669) or the viability (*p* = 0.303) between specimens from the younger and the older patients. However, in a study including liver pieces from as many as 1034 individuals, carried out in order to establish an algorithm that predicts the yield and viability of isolated hepatocytes, statistically significant differences between various groups have been found. After multiple regression analysis, apart from the tissue quality correlated with the disease, such variables as chemotherapy, sex, and age influenced the analyzed parameters. This study detected statistical significance in more variables than any other research due to the remarkable increase in the statistical power [7]. The authors indicated that their analysis was able to pick up a statistical significance where other studies found lack of interactions possibly because of a greater number of replicates. Moreover, they observed a loss of statistical power when multiple regression analyses were performed on the samples with reduced sizes (218 for viability and 128 for yield of the isolated cells). Thus, in our case, it is possible that the smaller number of analyzed tissue fragments was the reason for the lack of statistical significance in the liver cells’ yield and viability between the examined groups of patients.

In our report, median isolation efficiency and cell viability in the group of liver donors (n = 6) were lower than those obtained in the rest of the population (n = 35): 2.7 × 10^6^/g and 96.7% vs. 3.4 × 10^6^/g and 97.3%, respectively. These results, although not statistically significant (*p* = 0.209 and *p* = 0.19, respectively), are in line with others. Bhogal *et al*. (2011) in the study on isolation of the cells from 104 liver fragments reported lower viability and yield of hepatocytes from donor livers (n = 7) due to the longer cold ischemia time that donor livers have been exposed to when compared to normal livers [6]. In the research results presented by Hughes *et al*. (2006) cells isolated from fragments of the livers used for transplantation exhibited a significantly higher viability than those from livers rejected for transplantation (10 of 15 rejected livers were steatotic) [4]. Interestingly, our findings indicated that the donor livers not suitable for transplant due to the tissue quality also had lower percentage of Alb-positive P2 cells (77.9) than it was estimated for the remaining population (85.8), *p* = 0.458. Thus, it turned out that tissue quality played a major role in the successful isolation of healthy hepatocytes. Taking into account that donors group is not representative of the patients’ population (only males) we did not include it into the further statistical analysis.

In order to find the optimal source of human hepatocytes we have focused on the method that would enable us to estimate biosynthetic capacity of the isolated cells. We have developed a fast and reliable technique, based on flow cytometry, to analyze production of hepatocytes’ proteins within few hours after isolation. The method to estimate quality of the hepatocytes by the FACS analysis of the percentage of the Alb-positive cells in the P2 population is presented here for the first time. The idea is based on the assumption that using the given antibody one can detect target protein present in the cell in amounts above some threshold and that only certain levels of the albumin synthesis, characteristic of active hepatocytes, allow their detection. As it was shown for the parameters of cells isolation, patients’ characteristics such as age and chemotherapy, analyzed in the entire population individually, did not reveal statistically significant differences in the number of active hepatocytes (percentage of the Alb- and A1AT-possitive cells in the P2 population) in studied groups. On the other hand, the sex variable gave us satisfying statistical significance (*p* = 0.038) when cells were labeled with anti-Alb antibody. To our best knowledge, this report is the first to indicate, using FACS, that human male hepatocytes may be more productive in terms of albumin synthesis. Generally, we have found the anti-A1AT antibody less useful for our purposes. The reason for this could be explained by the characteristic of the particular antibody we have used for hepatocytes’ staining or by the differential expression pattern of these two proteins. Nevertheless, albumin is the major blood protein product of the liver. Difficulties in obtaining statistically significant differences between studied groups result from the relatively low size of the groups in combination with the broad range of the acquired data. Such a situation is typical for biological objects and more data are required to draw far-reaching conclusions. Nevertheless, based on the Alb staining, our results certainly indicated (*p*-values lower than 0.05) that: 1. samples obtained from males gave a greater percentage of active hepatocytes than those from females and 2. specimens from the males after chemotherapy greater than those from the treated females. In a large study on the cytochrome *P*450 enzyme activity in human liver microsomes (n = 142) and its inducibility in cultured human hepatocytes (n = 64) Parkinson *et al*. (2004) concluded that gender, age, ethnicity, and liver cirrhosis of the donor should not influence the selection of human liver samples for routine studies of drug metabolism and enzyme induction *in vitro*. The results indicated that the CYP enzyme activity varied considerably from one sample to the next and this variation was observed in human liver samples from males and females, young, middle-aged and elderly donors, and in livers from people of different ethnicity. A lack of statistical significance was apparent between analyzed groups (with some exceptions), yet the authors did not use combinations of these parameters for comparison [12]. In our opinion, regardless of the differences in studied hepatocytes’ activity markers, our analytical approach gives us a deeper insight into the problem. The results obtained in our study suggest that chemotherapeutic agents might affect albumin synthesis in male and female hepatocytes in a different manner. There is some evidence from clinical studies that physiological variables between human male and female hepatocytes are responsible for differential responses to drugs. In a more recent published study male and female hepatocytes were compared in terms of their susceptibility to drug-related hepatotoxicity and overall higher sensitivity of female primary hepatocytes was shown [13]. There is a large body of evidence that also age of the patient negatively affects physiological capacity of the hepatocytes. Aging impairs liver regeneration with respect to the number of hepatocytes that are able to enter S-phase after partial hepatectomy. It was shown that age-related hepatocyte telomere shortening might be responsible for this decline in cell proliferation (see [21] for a review). However, in our study this variable had no impact on the percentage of Alb-positive hepatocytes, even if we rearranged age ranges or performed multivariate analysis. Thus, the lowered proliferative capacity of the aged hepatocytes does not necessarily correspond to their biosynthetic potential.

In conclusion, our results show that there exists a complex dependence between the patient’s sex, chemotherapy treatment and the quality of the isolated hepatocytes in terms of their possible usefulness in a cell-based therapy and research. Both: females and males, older and younger people as well as patients who have undergone chemotherapy are all good sources of viable liver cells. On the other hand, livers that are not suitable for transplant are also not desirable in liver cells isolation. The age and the chemotherapy, when analyzed individually, did not affect the biosynthetic capacity of the hepatocytes (Alb and A1AT production). Some differences between groups were visible when certain combinations of these parameters were used. The most pronounced differences (and statistically significant) were observed between: males and females, and males and females after chemotherapy. The low number of samples is a major limitation of our report. For this reason, we have applied very restrictive statistical analysis, including tests for the correction for the variations of the sample size (see: Patients and Methods section), that allowed us to draw careful conclusions. Moreover, due to the low accessibility of such specimens for a research, many other papers report similar sizes of the analyzed samples or sizes of the same order of magnitude [4,6,11–13].

Although more samples are needed to draw firm conclusions we ranked sources of active hepatocytes by the median percentage of Alb-positive cells in the P2 population as follows:

Men under 60 years old (n = 7)– **92.6** (81.2–98.7)
Men over 60 years old (n = 11)– **85.8** (59.9–97.5)
Women under 60 years old (n = 11)– **80.3** (59.9–97.5)
Women over 60 years old (n = 6)– **79.8** (56.5–95.9)

## Supporting information

S1 Fig Interaction plot for term "Sex/Chemotherapy". (PDF)
