# Supplementary figures and images for: Liver tissue fragments obtained from males are the most promising source of human hepatocytes for cell-based therapies – Flow cytometric analysis of albumin expression
Source: PLoS One. 2017 Aug 9;12(8):e0182846. doi: 10.1371/journal.pone.0182846 (PMC5549982; doi:10.1371/journal.pone.0182846)

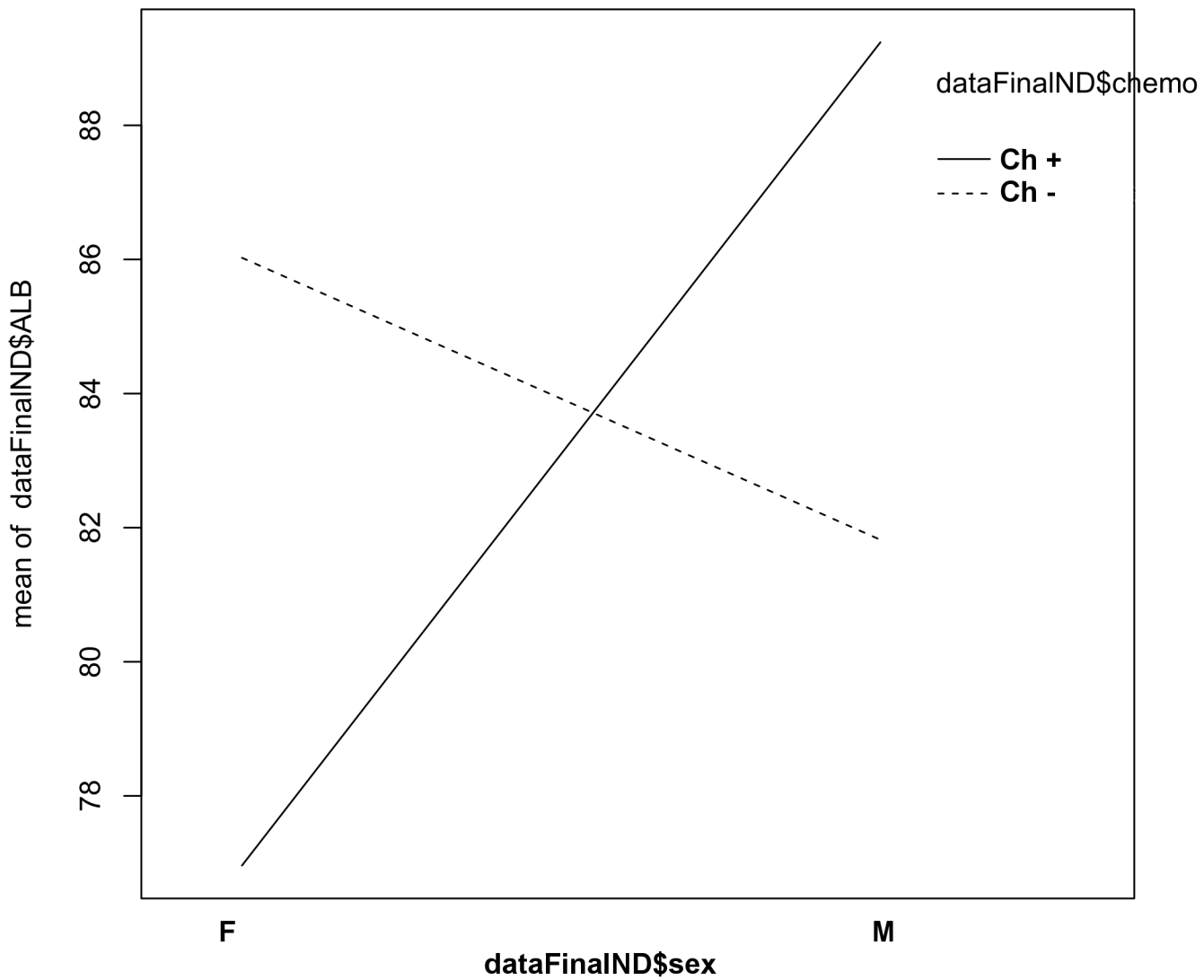

**S1 Fig.** Interaction plot for term "Sex/Chemotherapy".

Supplement: S1 Fig — (PDF) [file pone.0182846.s001.pdf]
